# Bridging Cost and Performance in Cutting Force Measurement: A PVDF-Based Universal Plate Dynamometer

**DOI:** 10.3390/s25216645

**Published:** 2025-10-30

**Authors:** Giovanni Totis, Alessandra Bordon, Federico Scalzo, Marco Sortino

**Affiliations:** Polytechnic Department of Engineering and Architecture, University of Udine, Via delle Scienze 206, 33100 Udine, Italy; alessandra.bordon@gmail.com (A.B.); federico.scalzo@uniud.it (F.S.); marco.sortino@uniud.it (M.S.)

**Keywords:** milling, cutting forces, dynamometer, PVDF, dynamics, filtering

## Abstract

Cutting force measurement plays a fundamental role in machining research and industrial applications, but existing dynamometers present important trade-offs between cost, stiffness, and dynamic bandwidth. Strain gauge devices are inexpensive but too flexible for high-speed operations, whereas piezoelectric systems provide excellent accuracy and bandwidth at prohibitive costs. This work presents the design, construction, and validation of a novel plate dynamometer based on polyvinylidene fluoride (PVDF) sensors, aimed at providing an effective alternative having an intermediate cost and suitable for advanced milling applications. The device integrates eight symmetrically arranged PVDF films in a stiff steel structure, complemented by four accelerometers for inertial compensation. A finite-element analysis confirmed favorable stress distribution at the PVDF contact surfaces and high resonance frequencies (under ideal clamping conditions). Modal tests demonstrated that uncompensated PVDF signals offer limited bandwidth, but the application of the Universal Inverse Filter (UIF) extended the usable bandwidth to 5 kHz along direct directions and up to 0.3–4 kHz along cross directions, approaching the performance of piezoelectric reference devices. Milling tests under diverse cutting conditions further validated the new device. Overall, the proposed device bridges the gap between low-cost strain gauge and high-performance piezoelectric dynamometers, offering a versatile and promising solution for both laboratory research and industrial applications.

## 1. Introduction

Cutting force measurements play a crucial role in machining, both in fundamental research and R&D activities, as well as in real industrial applications. Main purposes of the cutting force measurements include machinability testing and tool benchmarking [1,2], understanding cutting mechanics and dynamics [3], tool design and optimization [4], and process and tool condition monitoring [5]. Typical monitoring tasks involve tool breakage detection, remaining useful life estimation [6,7], and chatter detection [8]. Cutting force measurements are also essential for adaptive control of machine tools [9,10].

Currently, different types of devices and systems for cutting force estimation exist, developed over the last 40 years. According to the classification criterion proposed in [11], they can be roughly characterized as invasive or non-invasive, depending on their measurement principles and structural properties. Invasive devices have their own mechanical structure that must be embedded in the machining system chain. As a consequence, they alter the stiffness of the final machining system and can interfere with the working conditions. Their advantage is the higher sensitivity, accuracy, and reliability with respect to the non-invasive solutions.

In milling, plate dynamometers are invasive devices installed between the workpiece and the machine tool table, which perceive the cutting forces applied to the workpiece. In this case, the cutting forces are estimated from the inner deformation of the device. For this purpose, strain gauges can be used, which are very cost-effective but require a very flexible dynamometer structure to achieve the necessary sensitivity and an adequate signal-to-noise ratio [12,13,14] (See Table 1). As a consequence, they are generally too flexible and have a poor dynamic bandwidth, which is insufficient to investigate advanced milling applications that involve relatively small cutters rotating at high spindle speeds.

Additionally, piezoelectric plate dynamometers are very stiff and sensitive, showing highly repeatable linear behavior over a wider frequency range [15]. Their main drawback is their high cost, which hinders their applicability outside (well-equipped) research laboratories.

Other solutions have also been explored in recent years, employing different types of sensors, such as those based on optical principles, which nevertheless exhibited poor dynamic bandwidth [16] or too narrow a scope of application [17].

An interesting and underexplored alternative is represented by PVDF sensors, which are still based on the piezoelectric principle but embodied in a thin film of polymeric material. In comparison to classic piezoelectric load cells, the PVDF sensors are cheaper but still more expensive than strain gauges, while their characteristics are intermediate in terms of repeatability and signal-to-noise ratio [18,19].

The PVDF sensors have been successfully applied for the construction of promising cutting force measurement systems, mostly in the form of rotating dynamometers installed between the cutting tool and the spindle, which measure the cutting forces acting on the tool. The pioneering work in this direction was carried out by Li et al. in 1993 [20], who installed a single PVDF sensor behind the cutting insert of an inserted milling cutter, showing the potential of this solution. Different solutions were proposed by Ma et al. [21,22], who integrated the PVDF sensors in the cylindrical tool shank of a cemented carbide end mill with an external diameter of 25.4 mm. Eventually, Luo et al. in [23] designed an improved version of the device developed by Li in [20], by integrating three PVDF sensors behind each cutting insert of a multi-tooth cutter, showing the effective capability of three-dimensional measurement for each cutting edge. Unfortunately, these promising solutions have not been transformed into commercial products, likely due to their complexity and non-scalable nature.

Recently, Luo et al. in [24] integrated four PVDF sensors into a machine tool table prototype for measuring cutting forces in the working XY plane orthogonal to the main spindle axis. However, the simplicity of the design resulted in a poor dynamic bandwidth. In addition, such configuration was unable to measure the axial component $F_{z}$.

In this work, a novel plate dynamometer—incorporating the PVDF sensors—will be presented and designed to satisfy the following specifications:

- Capable of at least three-dimensional force measurement, in the range of about ±1 kN;
- Lower cost than the conventional piezoelectric dynamometers;
- Higher bandwidth—under ideal clamping conditions—than cost-effective devices, particularly compared to the strain gauge dynamometers;
- Higher stiffness than that of cost-effective dynamometers, i.e. comparable to that of conventional piezoelectric devices;
- Universal (machine-tool independent), with compensated bandwidth comparable to that of the reference piezoelectric device evaluated in [25].

**Table 1:** Comparison among the-state-of-the-art plate dynamometers and the proposed one.

Dyn. Ref.	Mini Dyn [15,25]	Delta Dyn [15]	This Work	[24]	[13]	[12]	[14]	[17]	[16]
Primary sensors	piezo.	piezo.	PVDF	PVDF	strain gauges	strain gauges	strain gauges	laser-opt.	optical
Sens. dir.	$F_{x,y,z}$ ($M_{x,y,z}$)	$F_{x,y,z}$ ($M_{z}$)	$F_{x,y,z}$	$F_{x,y}$	$F_{x,y,z}$	$F_{x,y,z}$	$F_{x,y,z}$	$F_{x,y}$ ($M_{z}$)	$F_{x}$
Range [kN]	0.25–1	≈1	≈1	<1	1	5	2	≈0.01	1.5
$h_{xx,yy}$ [$10^{-3}\mu$m/N]	≈4–9	≈7	≈3.0	not known	≈11	≈8.5	>10	≈1000	38
$h_{zz}$ [$10^{-3}\mu$m/N]	≈5	≈5	≈5.5	–	2.5	2	>10	–	–
$f_{n,1}$ [kHz]	≈5	≈4.7	≈3.8	<0.2	0.5	0.4	<1.1	≈0.1	0.8
Centr. mass [kg]	≈0.4	≈0.3	≈1	>1	8	16	>10	>1	≈1.2
Cost	↑↑↑↑↑↑	↑↑↑↑	↑↑	↑	↑	↑	↑	↑	↑

## 2. Design and Construction of the Device

**Figure 1:**
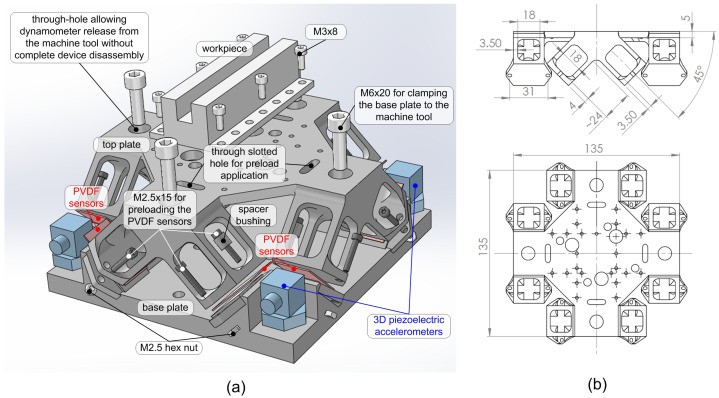
(**a**) Partially exploded view of the new device where the main elements are visible, including the PVDF sensors and accelerometers. (**b**) Main dimensions of the upper platform.

To meet the specifications outlined in the Introduction Section, the novel dynamometer was designed with the features shown in Figure 1 and described as the following:

- *Primary sensing elements*: eight PVDF sensors symmetrically arranged, theoretically capable of measuring $F_{x,y,z}$ and $M_{x,y,z}$. In this work, however, the focus is limited to the force components. Each PVDF sensor is pre-loaded using two steel M2.5 screws. The free length of each screw under pre-load, measured from the engaged fillets to the screw head, is approximately 9 mm, ensuring a sufficiently low elastic constant and, consequently, adequate sensitivity of the PVDF sensors.
- *Decoupling and force reconstruction*: the number and arrangement of PVDF sensors, together with the original design of the top plate, enable correct estimation and straightforward decoupling of all external load components ($F_{x,y,z}$ and $M_{x,y,z}$). Each force or moment excites either a subset or the entire set of sensors, inducing comparable pressures (possibly of opposite sign). Each PVDF sensor therefore contributes in a similar manner to the estimation of all force and moment components, in contrast to the behavior exhibited by the first and second prototypes of this device reported in [26] and [27], respectively, where distinct groups of sensors were dedicated to estimating specific force or moment components.
- *Minimization of shear stresses*: the PVDF sensors are designed to detect only pressure variations while being subjected to negligible tangential shear stresses, since excessive shear could damage the PVDF layers and increase cross-talk. This was achieved through a four-beam structure acting on each sensor (see Figure 1b), which is axially stiff but laterally compliant, thereby transmitting mostly normal pressure to the sensitive PVDF area. In the earlier prototypes presented in [26,27], the beams were smaller and more densely arranged, and could only be produced through additive manufacturing. In the present design, an equivalent effect is achieved with a simpler geometry that can be manufactured using conventional machining processes.
- *Mechanical structure*: the dynamometer consists of a base and a top platform with a symmetric design, both made of Ck45 carbon steel and entirely manufactured by conventional machining. This choice ensures moderate cost and high dimensional accuracy, with tolerances of about ±0.05 mm on functional surfaces.
- *Universality*: four triaxial piezoelectric accelerometers are mounted at the corners of the dynamometer base to monitor rigid-body motions. This enables effective compensation of inertial disturbances due to base vibrations—which are machine-tool-dependent, as demonstrated in [25]. In addition, through-holes in the top plate facilitate practical clamping and unclamping on any machine tool without requiring full device disassembly. This prevents loss of PVDF sensor pre-load and avoids the need for dynamic recalibration and recalculation of the Universal Inverse Filter.
- *Improved ergonomics*: the proposed design enables convenient assembly, straightforward sensor preload application, and unclamping of the dynamometer without complete disassembly—a limitation of the first prototype reported in [26].
- *Intermediate cost* (accelerometers excluded): the current approximate cost of a single PVDF sensor (of the type considered in this work), together with its dedicated cable and single-channel amplifier, is about EUR 500 per channel. By contrast, a high-end piezoelectric load cell with its dedicated low-noise, high-insulation cable and charge amplifier typically costs around EUR 2500 per channel. The four triaxial load cells of the reference dynamometer (Kistler Mini Dyn), together with their special cables and charge amplifiers—excluding the mechanical structure and other auxiliary components—amount to roughly EUR 30,000, since the system includes 12 channels in total. The complete commercial device, fully assembled and ready for use, costs at least twice as much. In comparison, the proposed device—including the PVDF sensors and their measurement chains but excluding the data acquisition system—can be estimated in the range of EUR 6000–12,000. Accordingly, the reference dynamometer lies between EUR 30,000 and 60,000, i.e., approximately five times higher. The proposed device thus achieves an intermediate cost: substantially lower than that of the reference piezoelectric dynamometer, yet inevitably higher than that of any strain-gauge-based solution, in line with the defined design specifications.

**Figure 2:**
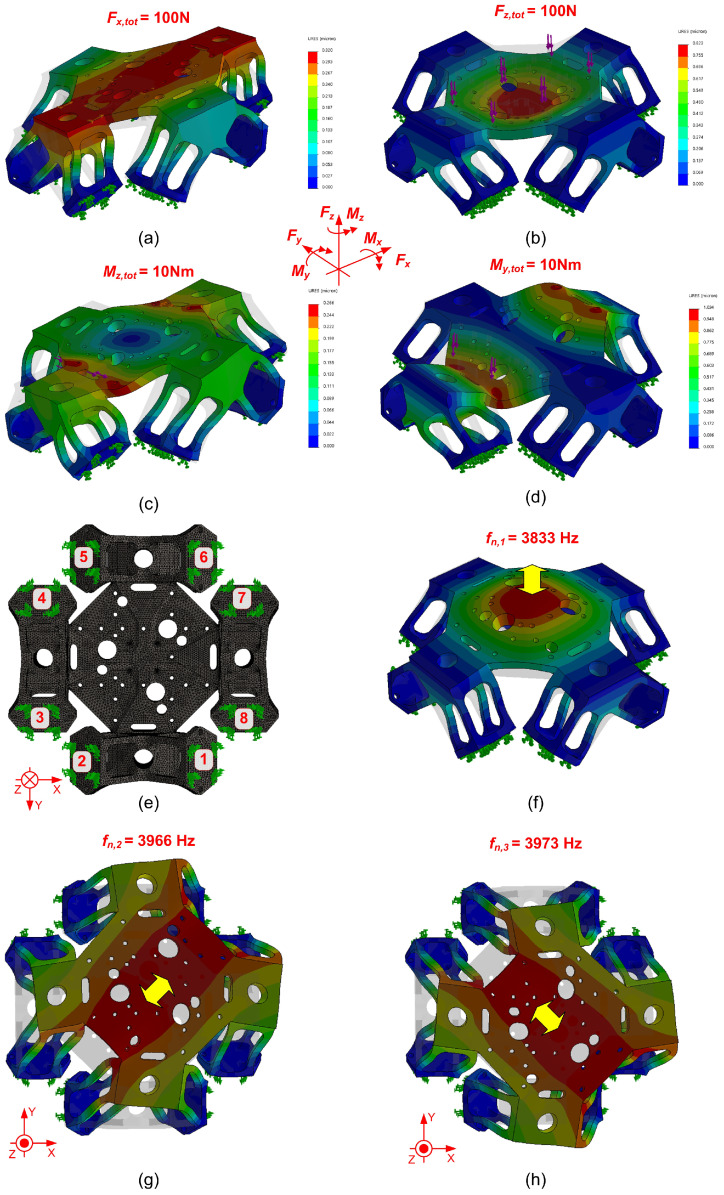
FEM analysis of the device to assess its static and dynamic behavior. Bottom view of the meshed component showing all PVDF contact surfaces, numbered from 1 to 8 (**e**), which are assumed to be perfectly clamped in all simulations. Results of static simulations under different loading conditions: transverse force (**a**), axial force (**b**), torsional moment (**c**), and bending moment around a horizontal axis (**d**). First three dominant vibration modes (**f**–**h**).

The mechanical behavior of the device was verified through a finite-element analysis (FEA) in SolidWorks Simulation (Dassault Systèmes, version 2015), using a curvature-based tetrahedral mesh with element sizes ranging from 1.5 mm to 0.3 mm, as shown in Figure 2e. This analysis enabled the numerical prediction of maximum deformation as well as the normal and shear stress distributions on the PVDF contact surfaces (where ideal clamping conditions were simulated) under different external loads, as illustrated in Figure 2a–c and summarized in Table 2. The ratio of tangential to normal forces on the most activated PVDF sensors was very favorable—below 10% for the transverse components $F_{x,y}$ and for all moments $M_{x,y,z}$, and only slightly higher for the axial direction $F_{z}$, though still within acceptable limits. Finally, the first natural frequencies of the top plate were simulated under the assumption of perfect constraints at the PVDF contact surfaces, yielding values higher than 3.8 kHz, as shown in Figure 2f–h. These frequencies are significantly higher than those of strain gauge dynamometers, which typically remain below 1 kHz.

**Figure 3:**
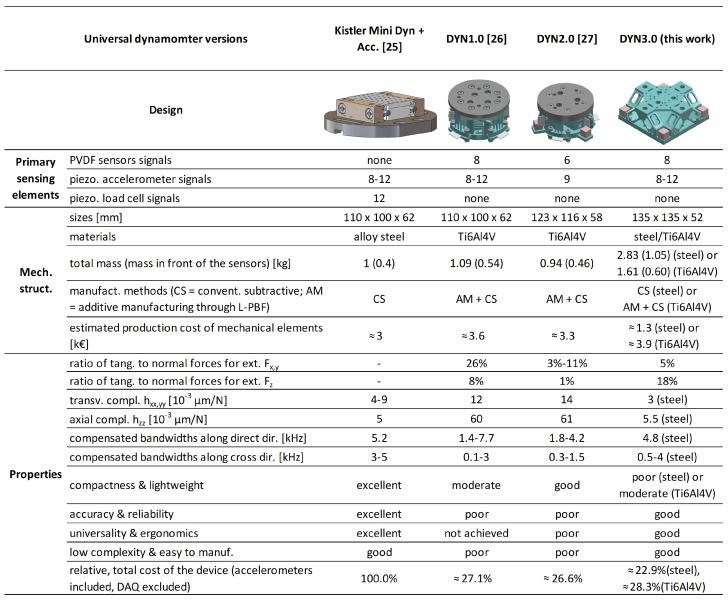
Comparison among the reference universal (piezoelectric) dynamometer developed in [25] and other recently developed three-dimensional dynamometers based on the PVDF technology, included the second prototype developed by Bordon in [27].

In Figure 3, a comprehensive overview is provided, contrasting the reference piezoelectric universal dynamometer described in [25] with all recently developed PVDF-based devices that can be regarded as three-dimensional platform dynamometers designed for universal use. The analysis considers the type of primary sensing elements, the mechanical structure, the main characteristics, and the estimated cost of each device. The results clearly highlight the superior features of the new device—representing the third prototype of the series—which will be further discussed in the following sections.

The PVDF sensor configurations embedded within the tooling system were excluded from the comparison above, as they differ substantially from the devices considered here. The only other independent PVDF-based plate dynamometer reported in the literature, proposed by Luo et al. in [23], was not included in Figure 3 because it is two-dimensional and not universal. It lacks integrated accelerometers and was not calibrated using the Universal Inverse Filter approach. However, a rough comparison between the Luo device and the newly proposed one is provided in Table 1, showing a considerably higher first natural frequency for the present device—typically associated with improved dynamic behavior—though at a slightly higher estimated cost, mainly due to the inclusion of accelerometers and the larger number of PVDF sensors.

From the synthetic comparison between the three prototypes reported in Figure 3, it can be noticed that the stress distribution on the PVDF sensors was significantly improved under $F_{x,y}$ loading, but not under $F_{z}$ loading. Nevertheless, the most distinctive feature of the new device is its considerably higher stiffness in all directions—comparable to that of the reference dynamometer and markedly superior to any strain gauge dynamometer developed so far, which requires a compliant structure to achieve adequate sensitivity.

One drawback is the relatively high mass of the top plate, manufactured in this case from Ck45 (approximately 1 kg). Using an alternative material such as Ti6Al4V could reduce the mass by about 45%, although at nearly three times the cost. This option has therefore been left for future investigation.

## 3. Experimental Setup and Configurations

**Figure 4:**
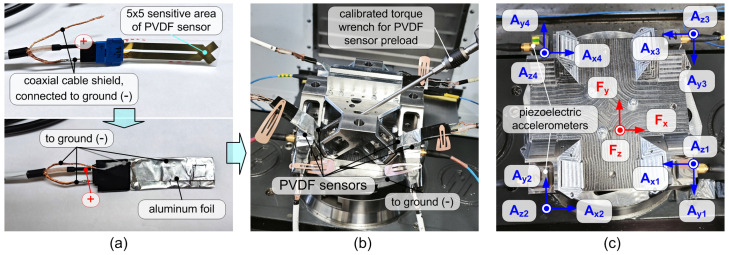
(**a**) Details of PVDF electrical insulation and connections, to minimize the noise. (**b**) Pre-load phase of the PVDF sensors using a calibrated wrench. (**c**) Accelerometers configuration.

In this work, Arkema–Piezotech Bauer Shock Gauges based on biaxially oriented PVDF (BO-PVDF) with a sensitive area of $5\times 5\;{\mathrm{mm}}^{2}$ were employed, visible on the top of Figure 4a [28].

Prior to integrating the sensing elements into the device, the electrical insulation of each PVDF sensor was performed with particular care to prevent unwanted disturbances from the external environment. For this purpose, the sensors were wrapped in a conductive aluminum foil, which was soldered to the ground of a coaxial BNC cable, as visible in Figure 4b. The insulated sensors were then connected to a precision differential charge amplifier (Vkinging VK10X, output ±5.5 V [29]), characterized by ultra-low noise amplification and low temperature drift. These precautions are essential to ensure correct acquisition of the PVDF signal.

Subsequently, each PVDF sensor and its measurement chain were individually tested using a Brüel and Kjær Type 4809 electromagnetic shaker in combination with a Brüel and Kjær Type 8230-001 piezoelectric load cell (sensitivity: 20.57mV/N) to assess their dynamic behavior. Data were acquired using a NI cDAQ-9178 system equipped with NI-9215 and NI-9234 modules. The sampling rate during these exploratory tests was 25.6 kHz or higher. The response of each PVDF sensor proved to be linear, repeatable, and coherent within the range of 60–6000Hz, although higher noise levels were observed outside this band, as reported in [26].

It should be recalled that the Universal Inverse Filter principle relies on the assumption that all sensor transmissibilities remain constant over time. Therefore, if significant sensitivity variations were to occur—either with time or due to other uncontrollable factors—they could compromise the development of a truly universal dynamometer based on the PVDF technology. To examine this aspect, additional experiments were carried out to evaluate the stability of the PVDF sensors. In particular, short- and medium-term tests were performed by re-measuring the dynamic transmissibility of a given PVDF sensor mounted on the shaker after different time intervals. Noticeable sensitivity variations were observed during the first few hours; however, after approximately four hours, the response stabilized and remained nearly constant over the following week, with measurements repeated once per day. A similar trend was found for other PVDF sensors, each exhibiting different transmissibility values–which was reasonably expected–but reaching a stable condition within about four hours. Consequently, all modal analysis and cutting tests reported in this work were performed at least four hours after the PVDF sensors’ pre-loading phase. Long-term stability tests were not carried out in this study, but they are considered important and should be addressed in future investigations.

For sensor pre-load, the base plate was rigidly clamped to the machine tool table. The top plate was then assembled, and each PVDF sensor was pre-loaded using a calibrated torque wrench (see Figure 4b) by applying a torque of approximately 7Nm.

Finally, four piezoelectric accelerometers (PCB 356A25, sensitivity: 25 mV/g), equipped with magnetic bases, were positioned as shown in Figure 4c. The accelerometers were connected to dedicated amplifiers featuring an anti-aliasing low-pass filter with a cut-off frequency of 10kHz, providing a voltage output of ±10V.

The dynamic calibration of the new device was performed through pulse testing. During modal analysis, the input forces were applied to the mechanical structure using an instrumented hammer (Dytran 5800B4, sensitivity 2.41 mV/N). In total, eight PVDF signals, twelve acceleration signals, and the hammer signal were acquired using an NI cDAQ-9178 system equipped with NI-9215 and NI-9234 modules. The sampling frequency was set to 25.6 kHz, and all data were processed in MathWorks MATLAB R2023b. The same DAQ configuration and sampling rate were also employed during the final cutting tests on the milling machine (although the hammer signal was, of course, absent).

**Figure 5:**
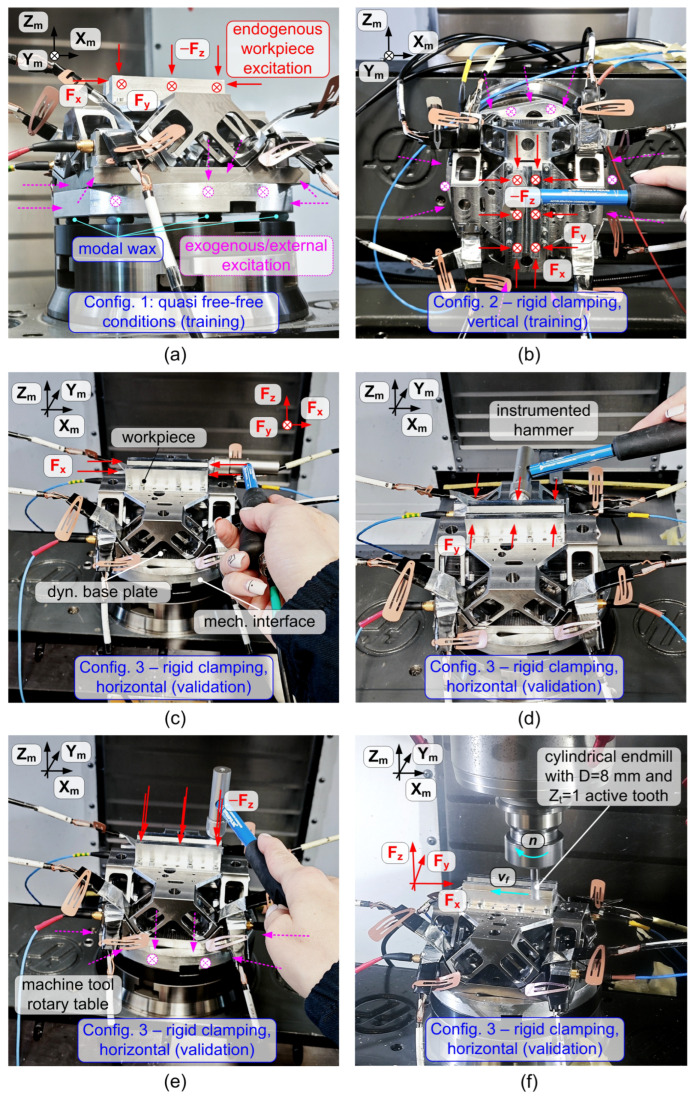
(**a**) Quasi free–free configuration used for training: the device is fixed to the rotary table of the machine tool using only modal wax. (**b**) Rigid clamping of the device on the rotary table, vertically oriented (rotations *A* and *B* both $90^{{}^{\circ}}$), also used for training. (**c**–**e**) Modal analysis in the validation configuration: the device is rigidly clamped on the rotary table, horizontally oriented (rotations *A* and *B* both $0^{{}^{\circ}}$), and impulsive forces are applied along *X*, *Y*, and *Z* directions, respectively. In all cases, both endogenous and exogenous pulse testing is carried out. (**f**) Experimental setup for cutting tests, performed in the final validation configuration.

To evaluate the performance of the device with and without filter compensation, the procedure described in [25] was adopted. The construction of the UIF filter required tests in three different dynamic configurations—two for training and one for validation—as illustrated in Table 3 and Figure 5.

Specifically, the dynamometer was tested under the following conditions, all performed on the HAAS VF2TR milling machine:

- Configuration 1 (quasi free-free): the device was attached to the machine tool table using only modal wax, enabling effective excitation of the dynamometer base (exogenous training);
- Configuration 2 (rigid, vertical and rotated): the device was rigidly clamped on the machine tool rotary table, which was vertically oriented and rotated; classic endogenous training (i.e. by hitting the workpiece) was mainly applied on this configuration;
- Configuration 3 (rigid, horizontal): the device was rigidly clamped on the horizontally oriented machine tool table, for final validation.

It is important to note that configurations 2 and 3, although performed on the same machine tool, differ due to the orientation of the rotary table, which was rotated by 90° around both the two available rotational axes. This was sufficient to cause a different dynamic behavior of the device.

To show this fact, it is necessary to recall the definition of direct transmissibility $T_{kk}\left(j\omega \right)$, given by the following equation:

(1) $$T_{kk}\left(j\omega \right)=\frac{R_{\mathrm{dyn},k}\left(j\omega \right)}{R_{k}\left(j\omega \right)}$$

where $R_{k}$ is the effective input force component acting along direction k=x,y,z while $R_{\mathrm{dyn},k}$ is the linear combination of the eight PVDF sensor signals—obtained from the quasi static calibration procedure, called RAW in the next section—representing the global force component measured by the dynamometer along the same direction.

**Figure 6:**
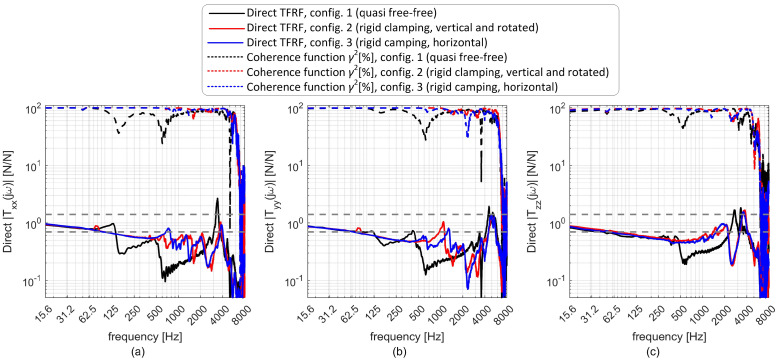
Direct transmissibilities derived from the PVDF sensors, showing the differences between the configurations used for training and that used for validation, along the *X* (**a**), *Y* (**b**) and *Z* (**c**) directions, respectively.

Direct transmissibilities obtained at the three different configurations are illustrated in Figure 6. While configuration 1 (free-free) is radically different from configurations 2 and 3 along all directions in the whole frequency range of interest, configurations 2 and 3 differ significantly at least along the *X* and *Y* directions in the range 0.5–4 kHz. This was considered sufficient to test the universality of the filter.

It is also worth noting that the dominant resonance peaks occur at around 4 kHz, in good agreement with the FEA predictions shown in Figure 2f–h and summarized in Table 1.

## 4. Modal Analysis

To assess the achievable performance of the device with different combinations of sensors and filters, the following filters were calculated and used, as also summarized in Table 3:

- **RAW**: Cutting forces reconstructed as simple linear combinations of the PVDF sensor signals (excluding accelerometers); the constant coefficients were derived from modal test data according to the quasi-static calibration procedure described in [11]; no further dynamic compensation was applied.
- **SOIF**: The Superior Optimal Inverse Filter [30,31] was computed using only PVDF sensor signals (accelerometers excluded) and trained in configuration 3 (the same used for validation) through classic endogenous excitation, i.e., by hitting only the workpiece.
- **UIF (auto-validated)**: The Universal Inverse Filter [25] was computed using both PVDF and acceleration signals and trained in configuration 3 (the same used for validation) through both endogenous and exogenous excitation, i.e., by first hitting the workpiece and then the dynamometer base, to train the device for generic rigid-body motions according to the UIF principles.
- **UIF (cross-validated)**: The Universal Inverse Filter [25] was computed using both the PVDF and acceleration signals and trained through endogenous and exogenous excitation in configurations 1 and 2, and validated in configuration 3, which exhibited a significantly different behavior, as discussed in the previous section. In this way, the capability of the new device to adapt to different experimental setups without a specific recalibration was verified.

**Figure 7:**
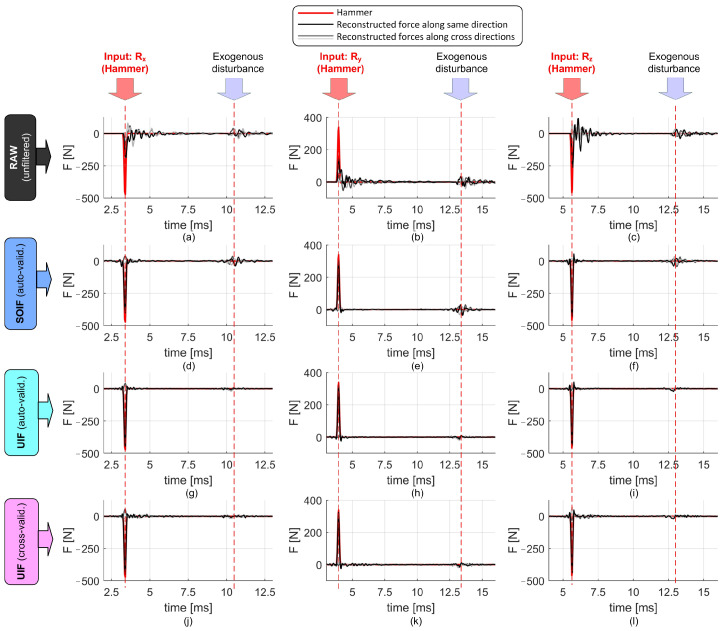
Raw and reconstructed impulsive responses obtained using different filtering techniques. (**a**,**d**,**g**,**j**): Input force applied along the *X* direction. (**b**,**e**,**h**,**k**): Input force applied along the *Y* direction. (**c**,**f**,**i**,**l**): Input force applied along the *Z* direction. For the cross-validated UIF, the input forces are accurately reconstructed along the correct directions, while cross disturbances remain low, as desired.

The comparison among effective input forces, raw, and reconstructed cutting force signals along both excited and cross directions is reported in Figure 7. While SOIF achieves a reasonably good reconstruction, the UIF approach provides superior filtering and attenuation of cross-talk disturbances, for both endogenous and exogenous excitations.

**Figure 8:**
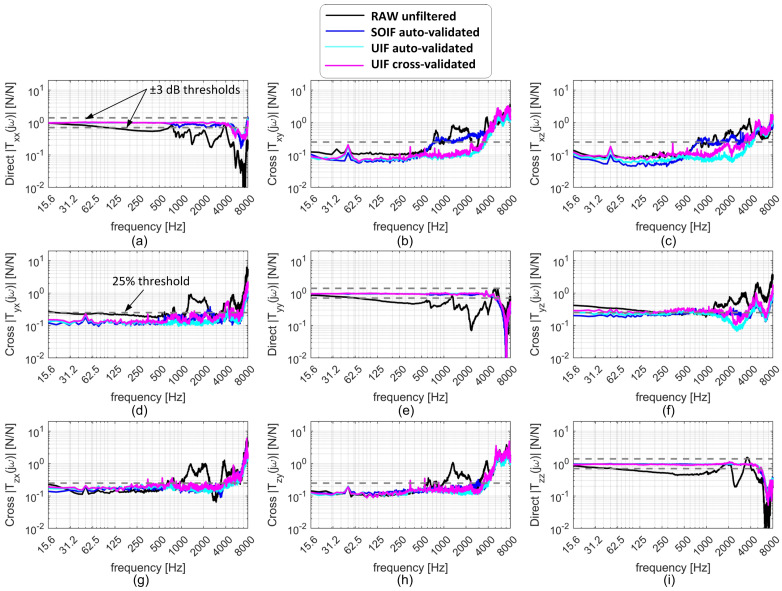
Transmissibilities of the device obtained with different filtering techniques, showing the satisfactory performance of the final cross-validated UIF filter compared with the reference SOIF (auto-validated in the final configuration without accelerometers) and the auto-validated UIF (the best, as expected). Along the direct directions (**a**,**e**,**i**), the compensated TFRFs should remain within the ±3 dB band, while along the cross directions (**b**–**d**,**f**–**h**) they should preferably stay below the 25% threshold.

Raw and compensated transmissibilities are presented in Figure 8. To quantify the performance of the various filtering strategies, the following criteria were adopted:

- The usable frequency bandwidth of the direct TFRFs was identified using the conventional ±3 dB limits relative to the ideal unit gain.
- The usable frequency bandwidth of the cross TFRFs was defined using a threshold of 0.25, considering that the ideal cross TFRF should be null.

**Figure 9:**
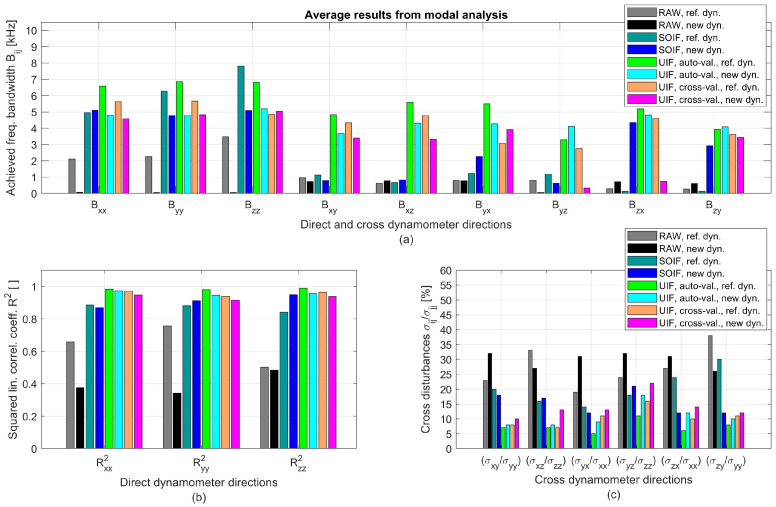
Dynamic performance of the novel PVDF-based device compared with the reference piezoelectric Kistler MiniDyn [25], as obtained from modal analysis. (**a**) Frequency bandwidths along the direct directions ($B_{xx}$, $B_{yy}$, $B_{zz}$) and cross directions ($B_{xy}$, $B_{xz}$, etc.). (**b**) Degree of linear correlation between the filtered signals and the reference hammer signal in the direct directions. (**c**) Cross-talk disturbances. Overall, the performance of the new device is comparable to, or only slightly lower than, that of the reference.

The resulting bandwidths for the direct and cross directions are reported in Figure 9a, where they are also compared with those obtained using the reference piezoelectric dynamometer presented in [25], applying the same filtering techniques. However, it should be recalled that the experimental configurations adopted for cross-validation were different in the two cases. The reference piezoelectric dynamometer was validated on a smaller and more flexible milling machine in [25], which was unfortunately not available for testing the new device proposed in this work. Therefore, a different rotary table orientation was adopted here to simulate the effects of an alternative setup, as described in the previous section.

The comparison is also illustrated in terms of the squared correlation coefficients between the real input signals (hammer) and the filtered responses (see Figure 9b). Finally, the cross-disturbances were quantified by $\sigma_{ij}/\sigma_{jj}\left[\%\right]$, defined as the ratio between the standard deviation of the filtered dynamic response along direction *i* and that of the hammer excitation along direction j≠i. These indicators are reported in Figure 9c.

From the analysis of these results, the following conclusion can be drawn:

- The dynamic bandwidth along the direct directions, without any dynamic correction, is very limited (about 100 Hz). This is most likely due to the intrinsic transmissibility of the PVDF sensors, which can vary over the entire frequency range, as already observed in preliminary shaker tests, reported in [26]. In addition, the vibration modes of the machine tool introduce inertial forces that disturb the uncompensated transmissibilities [11], even though the first mechanical resonance of the device (when clamped on an infinitely stiff base) is expected at about 4 kHz. Therefore, without correction, this PVDF-based device is mechanically stiffer but does not exhibit a significantly better bandwidth than a typical low-cost strain gauge dynamometer. Thus, without additional filtering of the PVDF signals, the advantage provided by the higher stiffness may not be sufficient to justify the higher cost of PVDF technology compared with strain gauge–based devices.
- By adopting the SOIF on the new device (without involving accelerometer signals and by validating the filter on the same configuration used for training), the dynamic bandwidth reaches approximately 5 kHz along the direct directions and 0.5–4 kHz along the cross directions. Therefore, PVDF-based devices such as the one proposed here can achieve good dynamic performance, provided that the available signals are processed through advanced, dedicated filters.
- By adopting the auto-validated UIF, the dynamic bandwidth reaches approximately 5 kHz along the direct directions and about 4 kHz along the cross directions. Similar results are obtained with the cross-validated UIF, confirming the universality property, although in some cross directions the dynamic bandwidth decreases to 0.3–0.8 kHz. As a result, some relevant cross-talk disturbances may be expected in the filtered cutting force signals.
- In general, the reference piezoelectric dynamometer exhibits slightly better performance, as expected. In particular, it was capable of effectively attenuating the cross-talk error, even when it was cross-validated on the noisier and more flexible milling machine considered in [25].

## 5. Cutting Tests

To prove the capabilities of the novel device under real cutting conditions, the dynamometer was tested during real milling tests, which were carried out on the configuration 3, by using Sandvik Coromant cylindrical endmill 2P230-0800-NA H10F, having *D* = 8 mm of external diameter, $Z_{t}$ = 1 active tooth, a negligible nose radius $r_{\varepsilon }\leq 0.1$ mm, normal rake angle $\gamma_{n}=13.5^{{}^{\circ}}$, and axial rake angle $\gamma_{a}=30^{{}^{\circ}}$.

Different cutting conditions were investigated following a fractional factorial design of experiments, as reported in Table 4. These included quasi-slotting, central milling of a thin-walled structure, and peripheral down- and up-milling tests, defined by different combinations of the lateral engagement parameters $a_{L1}$ and $a_{L}$.

The same conditions (identical machine tool, cutting tool, workpiece material, and cutting parameters) were also tested in [25] using the reference piezoelectric dynamometer. Those experiments yielded excellent agreement with the theoretical Shearing and Ploughing model presented in that work, whose mechanistic coefficients are recalled here for clarity (see Table 5).

For clarity, the main assumptions of the cutting force model are summarized below. Let us first assume that the cutting tool has a regular geometry (constant-pitch cutter) with $Z_{t}$ teeth, a uniform cutting edge angle $\chi =90^{{}^{\circ}}$, and an axial rake angle $\gamma_{a}=30^{{}^{\circ}}$. The nose radius $r_{\varepsilon }$ is initially neglected. We then consider a generic cutting edge element belonging to the *j*th tooth, whose position is defined by the feed motion angle $\varphi_{j}$ of the flute tip and by the axial coordinate *z* of the edge element (of infinitesimal length dz). The infinitesimal forces acting on such an element can be expressed as the following:

(2) $$\left\{\begin{array}{c} dF_{cj}≅g_{j}\left(k_{cs}dA_{j}+k_{cp}dz\right)\\ dF_{rj}≅g_{j}\left(k_{rs}dA_{j}+k_{rp}dz\right)\\ dF_{aj}≅g_{j}\left(k_{as}dA_{j}+k_{ap}dz\right) \end{array}\right.$$

where $dF_{cj}$, $dF_{rj}$, and $dF_{aj}$ denote the infinitesimal force components acting in the tangential, radial, and axial directions, respectively. The function $g_{j}$ represents the engagement condition of the cutting edge element with the workpiece, while $dA_{j}$ is the infinitesimal uncut chip area associated with that element. The parameters $k_{cs}$, $k_{rs}$, and $k_{as}$ [N/mm^2^] denote the shear coefficients, whereas $k_{cp}$, $k_{rp}$, and $k_{ap}$ [N/mm] are the ploughing coefficients in the corresponding directions, following [32].

The resultant force components are obtained by projecting the infinitesimal forces along the global (non-rotating) *X*, *Y*, and *Z* axes and summing the contributions over all flutes as the following:

(3) $$\left\{\begin{array}{c} R_{x}=\sum_{j=1}^{z_{t}}\int_{z=0}^{a_{p}}-dF_{cj}\cos\varphi_{j}-dF_{rj}\sin\varphi_{j}\\ R_{y}=\sum_{j=1}^{z_{t}}\int_{z=0}^{a_{p}}dF_{cj}\sin\varphi_{j}-dF_{rj}\cos\varphi_{j}\\ R_{z}=\sum_{j=1}^{z_{t}}\int_{z=0}^{a_{p}}dF_{aj}-\sum_{j=1}^{z_{t}}g_{j}\left(\varphi_{j}\right)k_{ar_{\varepsilon }}r_{\varepsilon } \end{array}\right.$$

where the axial component $R_{z}$ is corrected (for $z>r_{\varepsilon }$) to account for the effect of the nose radius $r_{\varepsilon }$ through an additional coefficient $k_{ar_{\varepsilon }}$, expressed in [N/mm]. It should be noted that the sign convention of all force components has been reversed in this work; therefore, the forces reported in the following figures correspond to those measured by the dynamometer, i.e., opposite in direction to the forces acting on the tool.

This model was eventually adopted as the main reference for evaluating the adequacy of the filtered cutting forces, since in this case no independent measured signal (such as the hammer signal in the modal analysis) was available. The same validation strategy was also employed in [25].

**Figure 10:**
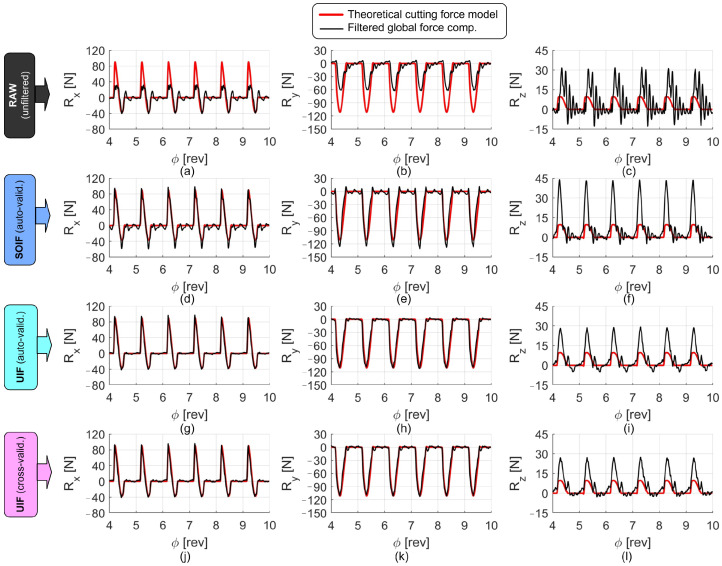
Comparison of the force components reconstructed using different filters under quasi-slotting milling conditions. Nominal cutting parameters: *n* = 15,000 rpm, $f_{z}$ = 0.1 mm, $a_{p}$ = 1.2 mm, $a_{L1}$ = 2.5 mm, $a_{L}$ = 6.5 mm. Raw forces are shown in (**a**–**c**). Forces reconstructed through SOIF are shown in (**d**–**f**). Forces derived from auto-validated UIF are illustrated in (**g**–**i**), while those obtained from cross-validated UIF are reported in (**j**–**l**).

**Figure 11:**
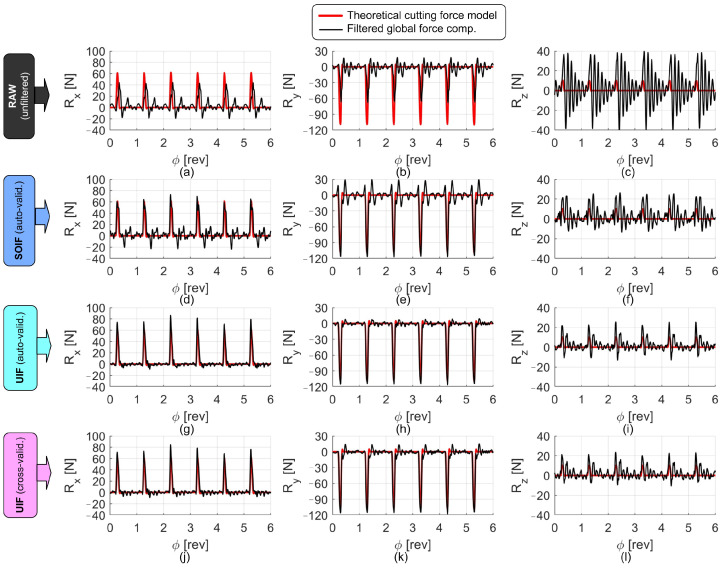
Comparison of the force components reconstructed using different filters during central milling of a thin-walled part. Nominal cutting parameters: *n* = 15,000 rpm, $f_{z}$ = 0.1 mm, $a_{p}$ = 1.2 mm, $a_{L1}$ = 1 mm, $a_{L}$ = 2 mm. Raw forces are shown in (**a**–**c**). Forces reconstructed through SOIF are shown in (**d**–**f**). Forces derived from auto-validated UIF are illustrated in (**g**–**i**), while those obtained from cross-validated UIF are reported in (**j**–**l**).

**Figure 12:**
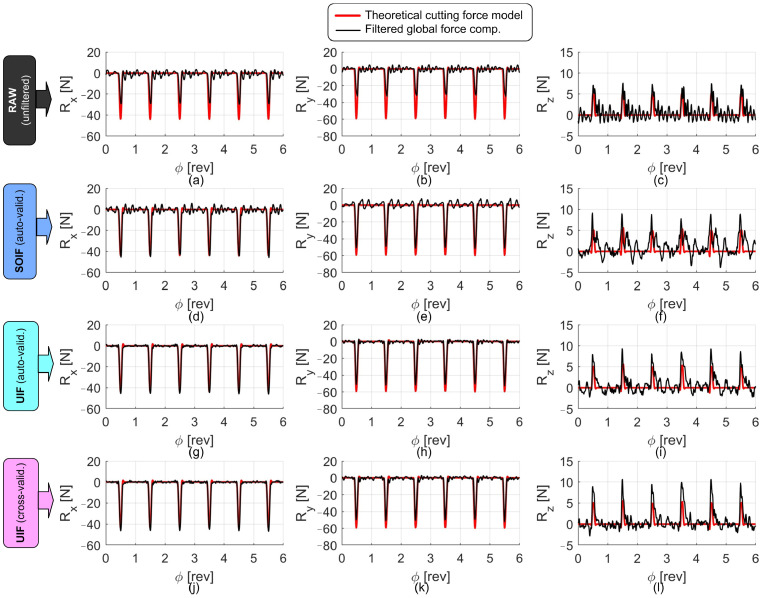
Comparison of the force components reconstructed using different filters during peripheral down-milling. Nominal cutting parameters: *n* = 15,000 rpm, $f_{z}$ = 0.05 mm, $a_{p}$ = 1.8 mm, $a_{L1}$ = −3.5 mm, $a_{L}$ = 0.5 mm. Raw forces are shown in (**a**–**c**). Forces reconstructed through SOIF are shown in (**d**–**f**). Forces derived from auto-validated UIF are illustrated in (**g**–**i**), while those obtained from cross-validated UIF are reported in (**j**–**l**).

Examples of the signals collected during the cutting tests, before and after the application of the filters, are shown in Figure 10, Figure 11 and Figure 12. In all figures, the reconstructed forces are compared with the theoretical ones obtained from the S&P cutting force model, developed on the same machine using the reference high-end piezoelectric dynamometer under identical cutting conditions (same tool, workpiece material, and cutting parameters). In all cases, the forces reconstructed through the UIF approach exhibit superior agreement with the model and a good level of correlation, except for the axial direction, where the actual input forces were relatively small and the measurements were therefore affected by a low signal-to-noise ratio and by cross-talk disturbances deriving from the other, more excited directions.

**Figure 13:**
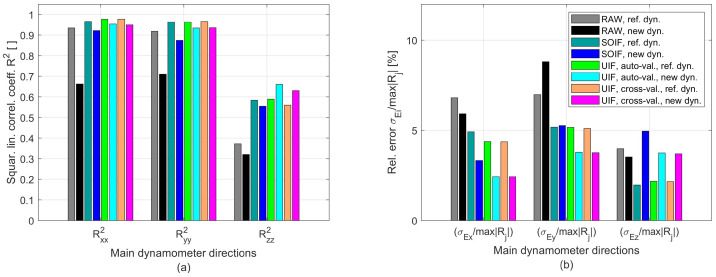
Comparison between the filtered cutting forces derived from real cutting tests, obtained from the novel device and the theoretical force model identified through the reference dynamometer in [25]. (**a**) Squared linear correlation coefficients between filtered force components and theoretical model. (**b**) Cross-talk disturbances normalized by the maximum force component.

From the analysis of all cutting tests (approximately 60), the indicators reported in Figure 13 were obtained. On the left-hand side, the squared linear correlation coefficients between the filtered and the theoretical forces $R_{ii}^{2}$, with i=x,y,z, are shown, whereas on the right-hand side the ratio $\sigma_{E_{i}}/\max\left|R_{i}\right|$ is presented. The latter quantity corresponds to the standard deviation of the absolute error between the model predictions and the filtered force in the *i*-th direction, normalized by its maximum absolute value $\max|R_{i}|$.

From the analysis of the cutting tests in the time domain, the following results were obtained.

The raw, uncompensated force signals deviate substantially from the real forces, as expected.

The forces reconstructed through SOIF (without accelerometer contributions) show moderately good agreement with the theoretical forces predicted by the reference model along the *X* and *Y* directions, but significant deviations persist along the axial *Z* direction. Therefore, when relying solely on the PVDF sensors as primary sensing elements and using a relatively massive top plate (about 1 kg, as in this case), the measurement capability can be considered adequate but not outstanding, particularly in the axial direction.

In contrast, the forces reconstructed through the auto-validated and cross-validated UIF show much better agreement across all directions. Nevertheless, the performance remains slightly worse on average along the *Z* direction, where notable cross-talk occurs. This behavior can be attributed to the cross-talk identified in the previous phase (e.g., the low $B_{zx}$ obtained with the cross-validated UIF) and to the fact that the input force components along the *X* and *Y* directions are considerably larger than the axial component, owing to the cylindrical geometry of the cutting tool. However, the main cause of this average behavior is likely the non-optimal stress distribution predicted by the FEA, where the ratio of tangential to normal stresses reached approximately 18% under an external axial load $R_{z}$.

## 6. Conclusions

This study has presented the complete design, construction, and validation of a novel plate dynamometer based on the PVDF sensors and accelerometers, aiming to provide a cost-effective and universal solution for cutting force measurement in milling. Main conclusions are drawn below.

First, the design choices adopted in the development of the device were proven successful. The novel sensor layout, with eight symmetrically distributed PVDF films, combined with a robust but conventionally machinable steel structure, has allowed the device to achieve the target balance between stiffness and sensitivity. Finite-element analysis confirmed that the transmission of loads to the PVDF elements occurs mainly through normal pressure, while the undesired shear stresses remain negligible or moderate, thus preserving sensor integrity and minimizing cross-talk. Furthermore, the predicted first resonance frequencies above 3.8 kHz, later confirmed by experimental modal tests, ensured that the mechanical structure itself does not represent the limiting factor in terms of bandwidth. However, its non-negligible mass of about 1 kg can affect the final performance of the device because of the associated inertial disturbances arising at the low-frequency resonance of the machine tool, unless a proper filter is applied for their correction. From a cost perspective, the device demonstrates a significant advantage over state-of-the-art piezoelectric dynamometers, while remaining considerably stiffer and more versatile—but certainly more expensive—than other typical strain gauge solutions.

Second, the modal calibration phase highlighted some potential issues related to PVDF stability and the intrinsic limitations of PVDF-based measurements when used without compensation. Specifically, it was observed that the static sensitivity and dynamic transmissibility of a single PVDF sensor mounted on a shaker can undergo noticeable variations after the pre-load phase; however, both quantities tend to stabilize after approximately four hours. Short- and medium-term stability tests confirmed that the transmissibility achieved after about four hours remained constant for at least one week. Nevertheless, long-term stability tests were not performed in this study and they should be addressed in future investigations. Moreover, the uncompensated PVDF transmissibilities revealed a very narrow usable bandwidth (on the order of 100 Hz), which is insufficient for modern high-speed machining research. This confirms that raw PVDF signals alone–without special filtering–cannot provide reliable dynamic measurements, especially in demanding milling scenarios. However, the subsequent application of advanced filtering techniques demonstrated the potential of postprocessing PVDF sensors signals through dedicated filters. In particular, the Superior Optimal Inverse Filter (SOIF) significantly improved the overall performance by exploiting only PVDF signals, while the Universal Inverse Filter (UIF)—integrating accelerometer data to correct for inertial disturbances—proved capable of extending the usable bandwidth to values comparable with those of reference piezoelectric systems (5 kHz along direct directions and 0.3–4 kHz along cross directions).

Third, when tested under real cutting conditions, the force signals reconstructed from the dynamometer measurements could be directly compared with the theoretical forces predicted by the Shearing & Ploughing cutting force model identified through the reference piezoelectric dynamometer. Filtered forces obtained from SOIF yielded reasonable agreement in the feed and cutting directions, but still showed large discrepancies in $F_{z}$. By contrast, UIF-based reconstructions produced far better consistency with the theoretical reference across all directions. Some degradation was still observed in the axial component, mainly due to the low magnitude of axial forces in the considered end-milling tests and the resulting susceptibility to cross-talk from the much larger lateral force components.

This study demonstrates that a PVDF-based plate dynamometer, when combined with advanced filtering, can approach the performance of high-end piezoelectric devices at a fraction of the cost. The prototype meets the intended targets of stiffness, compensated bandwidth, and universality, making it a valuable alternative to strain gauge or other low-cost solutions, especially for laboratories and industries unable to invest in conventional piezoelectric systems. While piezoelectric dynamometers still offer superior dynamic performance and robustness to cross-talk, the gap has been significantly narrowed.

Future work will focus on reducing the mass of the top plate (for example by producing it of Ti6Al4V, which is lighter but much more expensive), testing low-cost accelerometers, and evaluating the long-term reliability of PVDF sensors.

## Figures and Tables

**Table 2:** Forces perceived by the PVDF sensors under different loading conditions. An external force of 100 N was chosen arbitrarily—due to the linearity of the FEA approach—although the device was designed to measure loads up to 1 kN. Green rows correspond to the most loaded PVDF sensors, which are characterized by a moderate or small ratio between tangential and normal stresses.

Loading Condition	PVDF #	Ext. Load Adsorption	Mean Normal Force $|F_{n}|$ [N]	Mean Tang. Force $|F_{t}|$ [N]	Mean Ratio $|F_{t}/F_{n}|$
$F_{x}=100$ N	1,2,5,6	**89%**	**30.3**	**1.4**	**5%**
	3,4,7,8	11%	1.4	2.8	211%
$F_{z}=100$ N	1–8	**100%**	**17.1**	**2.9**	**18%**
$M_{z}=10$ Nm	1–8	**100%**	**32.3**	**1.2**	**4%**
$M_{y}=10$ Nm	1,2,5,6	10%	7.5	2.4	32%
	3,4,7,8	**90%**	**74**	**7.4**	**10%**

**Table 3:** Overview of the filters applied in the performance assessment of the new dynamometer relative to the reference dynamometer.

Dynam.	Filter	Use of	Training					Valid.
**Type**	**Type**	**Accelerom.**	**Config. 1**	**Config. 2**	**Config. 3**	**Endog.**	**Exog.**	**Config. 3**
Piezoel. Kistler Mini Dyn	RAW	–			√			√
	SOIF	–			√			√
	UIF (auto-val)	√			√	√	√	√
	UIF (cross-val)	√	√	√		√	√	√
New PVDF Dyn	RAW	–			√			√
	SOIF	–			√			√
	UIF (auto-val)	√			√	√	√	√
	UIF (cross-val)	√	√	√		√	√	√

**Table 4:** Design of experiments for cutting tests.

Factor	Levels	Values
Spindle speed *n* [rpm] (cutting speed $v_{c}$ $\left[m/\min\right]$)	2	10,000 (251), 15,000 (377)
Feed per tooth $f_{z}$ [mm]	2	0.05, 0.1
Depth of cut $a_{p}$ [mm]	3	0.6, 1.2, 1.8
Lateral tool-workpiece immersion ($a_{L1},a_{L}$) [mm]	4	(2.5, 6.5), (−3.5, 0.5), (4, 0.5), (1,2)

**Table 5:** Cutting force model coefficients estimated by the reference dynamometer in the same experimental setup (same machine tool, cutting tool and workpiece) [25].

$k_{\mathrm{cs}}$ [MPa]	$k_{\mathrm{rs}}$ [MPa]	$k_{\mathrm{as}}$ [MPa]	$k_{\mathrm{cp}}$ [N/mm]	$k_{\mathrm{rp}}$ [N/mm]	$k_{\mathrm{ap}}$ [N/mm]	$k_{{\mathrm{ar}}_{\varepsilon }}$ [N/mm]
725.8	290.2	87.1	21.9	19.6	3.8	40.2

## Data Availability

The data supporting the findings of this study are not publicly available due to ongoing patenting procedures and related intellectual property protection. Data may, however, be made available from the corresponding author upon reasonable request and subject to the authors’ discretion, once confidentiality constraints have been lifted.
